# Effect of different restorative crown design and materials on stress distribution in endodontically treated molars: a finite element analysis study

**DOI:** 10.1186/s12903-020-01214-3

**Published:** 2020-08-18

**Authors:** Jie Lin, Zhenxiang Lin, Zhiqiang Zheng

**Affiliations:** 1 Fujian Key Laboratory of Oral Diseases, School and Hospital of Stomatology, Fujian Medical University, 246 Yangqiao Zhong Road, Fuzhou, Fujian 350002 People’s Republic of China; 2 Department of Crown and Bridge, School of Life Dentistry at Tokyo, The Nippon Dental University, 1-9-20 Fujimi, Chiyoda-ku, Tokyo, 102-8159 Japan; 3 Department of Stomatology, Hospital of Fujian Provincial Authorities, 68 Guping Road, Fuzhou, Fujian 350001 People’s Republic of China

**Keywords:** Finite element analysis, Endocrown, Post-core crown, Fiber post, Zirconia

## Abstract

**Background:**

The purposes of this simulation study were to evaluate the stresses in the roots of endodontically treated molars with extensive coronal tissue loss which were restored by endocrowns (all-in-one core and crown) and traditional crowns with post-cores, during masticatory simulation using finite element analysis.

**Methods:**

A mesio-distal cross-section of a lower right first molar was digitized and used to create 2-dimensional models of the teeth and supporting tissue; different crown designs, viz., endocrown with 2 mm occlusal clearance, endocrown with 4 mm occlusal clearance and post-core crown; different crown materials, viz., zirconia (Zr) and lithia-disilicate reinforced glass ceramic (LDRGC), and different post and core materials, viz., glass fiber (GF), stainless steel (SS) and metal cast (MC). An axial load of 600 N was applied to the central fossa of occlusal surface.

**Results:**

The stress distributions were similar between Zr and LDRGC for periodontal ligament and alveolar bone. The root canal inner wall maximum principal stresses of SS post (70.8 MPa) and MC post (71.4 MPa) were higher than that of GF post (36.0 MPa) and endocrown (2.4 MPa).

**Conclusion:**

The endocrowns reduced stress concentration for the root canal inner wall in comparison with the conventional post-core crown. Molars restored with endocrowns are less prone to root fracture than those with posts.

## Background

The decision of how to rehabilitate endodontically treated molars (ETM) with extensive loss of coronal structure is a challenge in restorative dentistry. Coronal tooth tissue is often significantly damaged after endodontic treatment and are traditionally restored with metal posts and cores and prosthetic crowns [1–3]. Initially, it was believed that this procedure would provide better reinforcement of the remaining dental structure [4, 5]. However, it has been observed that the use of intracanal retainers only help in the retention of the prosthetic crown. As a result of removing a dental structure to enable the placement of rigid dental materials devoid of mechanical behaviors similar to those of the tooth, the remaining tooth is weakened [6]. The preparation of a molar for a post in relatively narrow root canals also involves a risk of accidental root perforation and fracture [7].

In fact, minimally invasive preparations, with maximal tissue conservation, are now considered ‘the gold standard’ for restoring ETM [8]. In 1995, the endocrown was described by Pissis who is the forerunner of the endocrown technique, as the ‘mono-block porcelain technique’ [9]. Currently, due to the advances in adhesive methods and materials, endocrown type of intracoronal restorations were suggested for damaged posterior teeth as an alternative to post and core retained ones [10]. It is a method particularly indicated in cases in which there is excessive loss of hard tissues of the crown, interproximal space is limited, and traditional post-core crown is not possible because of inadequate ceramic thickness [11]. Their advantages include the fact that tooth structures require little preparation, ease of preparation, demand less clinical time when compared with conventional crowns [12, 13].

Knowledge of the stress distribution within and around the roots is a key factor for understanding root fracture, which are well-known problems with ETMs. It has been proposed that molars restored with endocrowns are less prone to fracture than those with posts [14, 15]. Nevertheless, so far there was no clear evidence to prove it. Some studies discussed the effect of preparation designs on the performance of endocrown. Tribst et al. [16] showed in finite element analysis (FEA) study that the stress is more concentrated on the restoration more than the cement line, if the remaining dental tissue is bigger and if the material has high elastic modulus. Dejak et al. [17] compared equivalent stresses in molars restored with endocrowns as well as posts and cores during masticatory simulation using FEA and found the tensile stresses achieved were 4 times higher values than under endocrowns. These tensile stresses occurred in the dentin under the crown shoulder, rather than in the root. Dejak’s study described the stress distribution for the crown in detail, while the current study focused on the stress distribution for the root. A similar study by Lin et al. [15] showed that the stress values on the dentin and luting cement for the endocrown restoration were lower than those for the crown. However, these studies made no attempt to compare endocrowns and post-core crowns, relatively little is known about the differences of stress distribution in the roots.

The type of restoration (endocrown or post-core crown, different crown and post materials) will provide rational stress distribution and reduce a risk of fracture in molars? Because of the absence of information about the biomechanical behavior of endocrowns and the expectation that this type of restoration would behave similarly or superiorly to post-core crowns, the present study has aims to evaluate maximum principal stress (MPS) in the roots of ETMs with extensive coronal loss, restored by endocrowns and post-core crowns, during masticatory simulation using FEA, and simulate stresses at the first molar made with different crown and post materials.

## Methods

The research protocol was reviewed and approved by the Research Ethics Committee at the School and Hospital of Stomatology, Fujian Medical University (No. 2017-CX-12).

### Structures and geometric conditions of the computer aided design (CAD) model

**Fig. 1:**
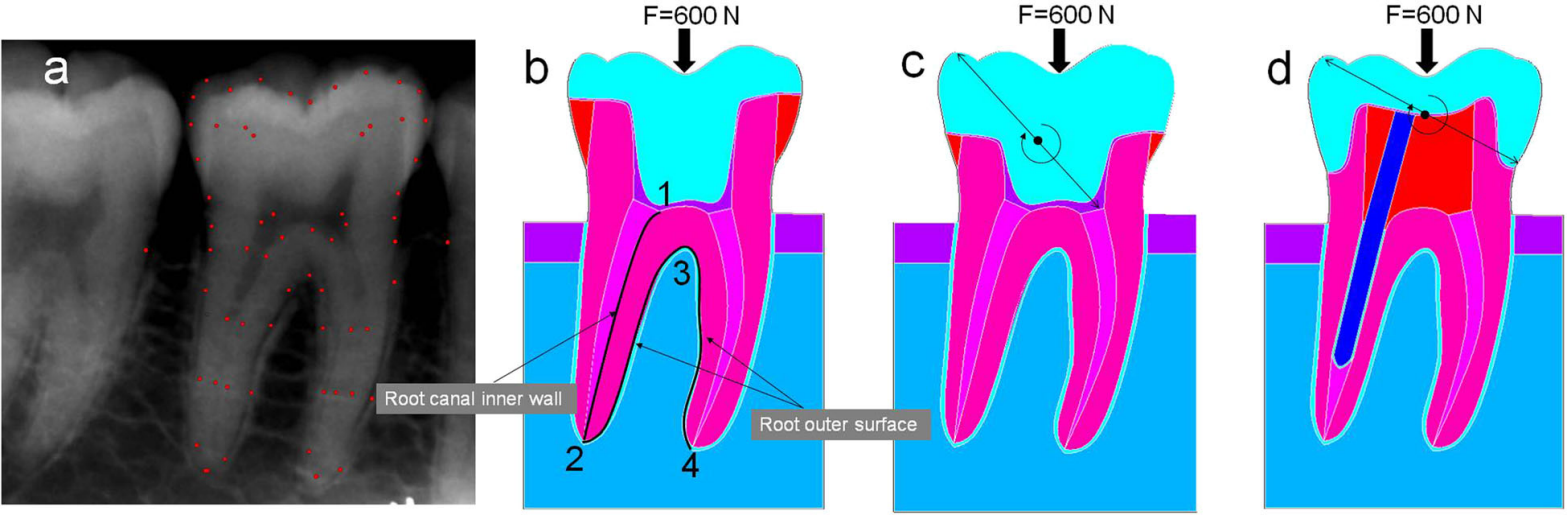
2D models of first mandibular molar tooth with roots and periodontium (bucco -lingual side view). Original contours developed from radiographic image of first mandibular molar tooth. **a** radiographic image of first mandibular molar tooth, the coordinate values at the red point were extracted. **b** endocrown with 2 mm occlusal clearance, (**c**) endocrown with 4 mm occlusal clearance. **d** post-core crown Thick arrow: simulated 600 N vertical occlusal load. 1 → 2: distal root canal inner wall, 2 → 3 → 4: root outer surface. Endocrown geometrically reduced the rotation center of the crown restoration in comparison with the full crown

A periapical film of the lower right first molar was taken by paralleling technique (Heliodent Plus, Sirona Dental Systems, Bernsheim, Germany) to generate a two dimensional (2D) image file (jpg). Microsoft Office PowerPoint 2007 (Microsoft, USA) was used to trace the points on the X-ray film (Fig. 1a), recorded the coordinate values of red points. The coordinate values of these points are imported into a FEA software (ANSYS v. 10; ANSYS Inc., Canonsburg, PA, USA) through ANSYS parametric design language (APDL) to create the 2D model.

**Fig. 2:**
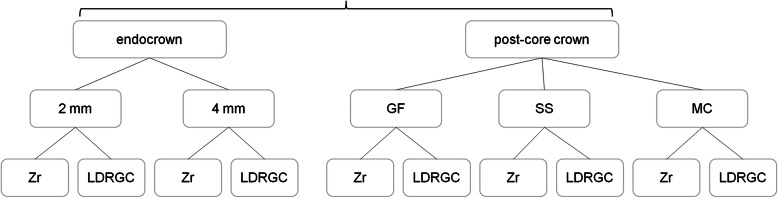
The flowchart of this study

There were three different model designs (Fig. 1), viz., endocrown with 2 mm occlusal clearance, endocrown with 4 mm occlusal clearance and post-core crown. The restorations used two different crown materials, viz., zirconia (Zr) and lithia-disilicate reinforced glass ceramic (LDRGC), and three different post and core materials, viz., glass fiber (GF), stainless steel (SS) and metal cast (MC). There were ten kinds of combination in this study. Detailed flowchart showing the group distribution has been included in Fig. 2. In the GF posts and SS posts, the cores were made of composite, while in the MC posts they were made of metal.

### Endocrown and post-core crown designs

The Endocrown-2 mm designs were created with 2.0 occlusal clearance, 7.0 mm cavity depth, and 5.3 mm base width. The prepared cavity walls tapered with 6–8 degrees from the cavity base to the cavosurface (Fig. 1b). The Endocrown-4 mm designs were created with 4.0 occlusal clearance, 5.0 mm cavity depth, 5.3 mm base width and 6–8 degrees cavity walls taper (Fig. 1c). Jacket crown preparations were created with 2.0 mm occlusal clearance, 0.5–1.5 mm cervical clearance and shoulder margin, 6–8 degrees tapering angle for first molars, 14.0 mm post lengths. Rounded shoulder margins and anatomic occlusal reduction were incorporated in model (Fig. 1d).

The surrounding bone was modeled as cortical bone (1.5 mm thickness) and cancellous bone, which were assumed to be isotropic, homogeneous, and linearly elastic. A 0.2 mm periodontal ligament [17, 18] was modeled around the roots. A 0.1 mm thick cement-imitating layer [17, 18] was formed around the root part of the created post and under the crown. A small gap of cement was added to the bottom of the endocrown cavity, which was closer to the clinical situation. Perfect bonding was assumed at all the interfaces, including those between the teeth, the cores, the crowns, the posts and bones.

### Material properties, mesh generation and boundary conditions

The Young’s modulus and Poisson’s ratios of the materials used are shown in Table 1 [18–26]. Material properties were assumed to be isotropic, homogenous, and linear-elastic, except the GF post. The material of GF post was anisotropic (Young’s modulus along its long axis was 38.5 GPa, and 12.0 GPa perpendicular to that axis).

**Table 1:** Properties of restorative materials and tooth tissue

	Young’s modulus (GPa)	Poisson ratio
Enamel [18]	84.1	0.33
Dentin [19]	18.6	0.32
Gutta-percha [20]	0.14	0.45
Periodontium [18]	0.15	0.45
Cortical bone/ Cancellous bone [21]	13.7/ 1.37	0.3
Luting resin cement [18]	7.5	0.3
LDRGC [22]	96	0.23
Zirconia [18]	200	0.31
Glass fiber post: Fiber_longitudinal/ Fiber_transverse [23]	38.5/ 12	0.35/0.11
Metal cast post (Ni-Cr) [24]	188	0.33
Stainless steel post [25]	210	0.3
Composite resin core [26]	7	0.3

LDRGC: lithia-disilicate reinforced glass ceramic

For calculation purposes, each tooth model was divided into 2D 4-node structural solid elements (PLANE42). This element is defined by four nodes having two degrees of freedom at each node: translations in the nodal x and y directions. In model with endocrown-2 mm, 30,158 elements joined at 30,318 nodes were used. In model with endocrown-4 mm, 29,663 elements joined at 29,821 nodes were used. In model with post-core crown, 29,847 elements joined at 30,002 nodes were used. The aim of this preliminary FEA was to identify the regions with highest stress concentration within the restoration, especially those along the distal root inner and outer surface. These would be the regions to which shape optimization would be applied. Thus, the mesh around the distal root inner and outer surface was made much finer than those in the other areas, with an average element edge length of 0.05 mm.

Fixed zero-displacement in both the horizontal and vertical directions was defined at the horizontal and vertical cut-planes of the supporting bone. An axial load of 600 N [18, 27] was applied to the central fossa of occlusal surface. MPS values were calculated by FEA along the distal root canal inner wall and the root outer surface (Fig. 1b: 1 → 2 → 3 → 4). This FEA study focused on the distal root canal inner wall because the post was set in the distal root canal, from preliminary analysis the distal root canal inner wall was analyzed in greater detail. The stress distribution within the tooth/restoration cross-section was solved with the FEA software (ANSYS).

## Results

**Fig. 3:**
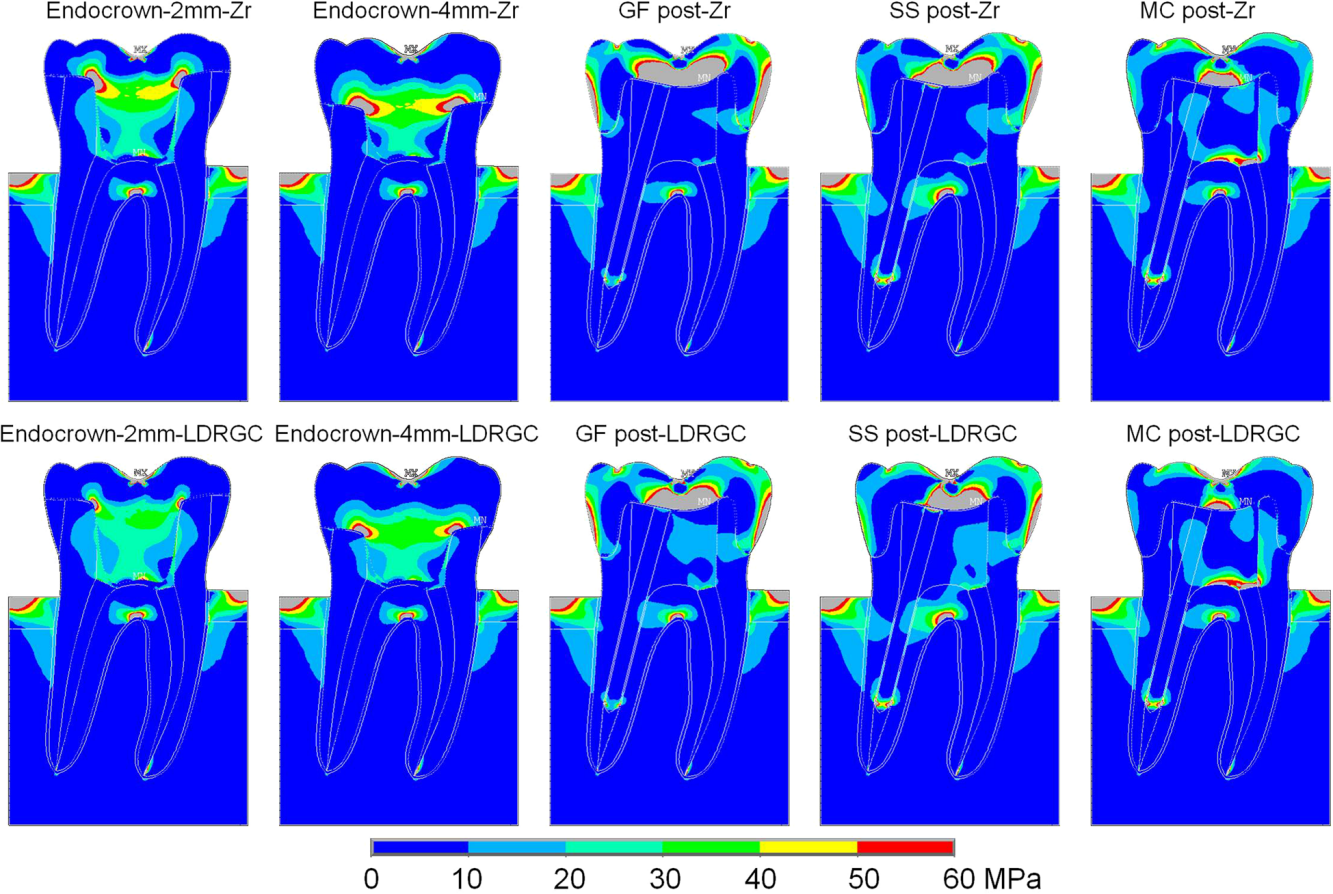
The MPS distribution maps in FEA results. Color bar indicates range of 0 to 60 MPa

MPS analysis for the 2 crown designs, 2 crown materials, 3 post materials tested is presented in Fig. 3. For periodontal ligament and alveolar bone, the stress distributions were similar between Zr and LDRGC. For the crown, Zr demonstrated higher and wider range MPS than LDRGC. For the cement layer, crown materials and the thickness influenced the stress distribution, endocrown-2 mm (Zr 31.7 MPa, LDRGC 35.9 MPa) demonstrated higher MPSes than endocrown-4 mm (Zr 27.2 MPa, LDRGC 31.6 MPa), Zr showed lower MPS than LDRGC.

**Fig. 4:**
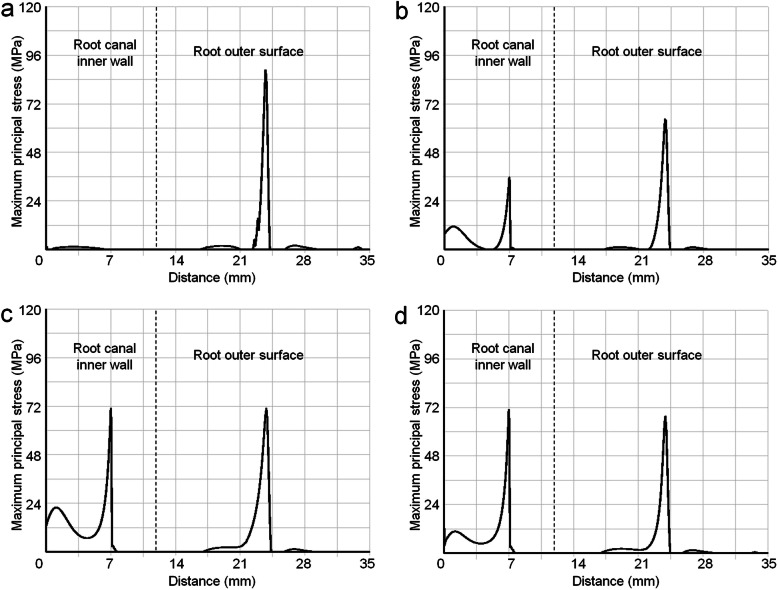
The path plots of MPS along the distal root canal inner wall (left side, 1 → 2) and the root outer surface (right side, 2 → 3 → 4). **a** All types of endocrown; **b** GF post; **c** SS post; **d** MC post. The left peaks in **b**, **c** and **d** were caused by the post tip, the right peaks were caused by root furcation area

Figure 4 shows the path plots of the interfacial MPS along the distal root canal inner wall and the root outer surface in endocrown and post-core crown. No difference was found in the Zr crowns and the LDRGC crowns in the path plots. In the endocrown covered tooth, MPS values of 2.4 MPa were recorded in the distal root canal inner wall. The root canal inner wall stresses of SS post (70.8 MPa) and MC post (71.4 MPa) were higher than that of GF post (36.0 MPa) and endocrown (2.4 MPa). The behavior of the endocrown clearly differed from that of the post-core crowns. The endocrown only had one peak (Fig. 4a), nevertheless the post-core crowns had two peaks (Fig. 4b, c and d). The peaks were caused by the root furcation area and the post tip. In the root furcation area, MPS of endocrown achieved a value of 88.7 MPa. The endocrown showed higher stresses occurred in the root outer surface than that of post-core crowns.

## Discussion

The numeric FEA modeling is able to reveal the otherwise inaccessible stress distribution within the tooth-restoration complex. It has proven to be an important tool in the design process for the understanding of tooth biomechanics and the biomimetic approach [28]. It is undeniable that the 2D model has its limitations. It can only reflect a simplified plane, and can not fully reflect the three dimensional (3D) structure of teeth, which is not complete in mechanics. The focus of this study was root fracture and stress distribution, so it needed a clear and definite conclusion that the vertical force of the molar with two roots. Although teeth are 3D structures, important mechanical events in endocrown and post-core crown appear within the mesio-distal plane [29]. 2D abstraction is a highly generalization of the nature of 3D problems. There were many successful applications of 2D finite element in dentistry in the past five years [23, 30]. These events support the use of the 2D plane-strain model for numerical analyses. Volume meshing of 3D teeth structures is usually restricted to tetrahedral elements. The tetrahedral element has a good ability to model irregular shapes; however, its accuracy is poor for bending and shearing dominated problems. A convergence test is always needed to be conducted to determine the size of elements in finite element modeling. In this 2D FEA, the mesh was refined continuously until the FEA result did not change greatly. Interface contact analysis was not involved in this study. In contacting surfaces and irregular 3D model usually there are singular points tolerating maximum sharp peak stress. The use of a 2D model is also valuable because of its improved performance in terms of element number and simulation quality.

The stress distribution depends strongly on the functional loads in FEA study. The axial load of 600 N was to simulate the maximum bite force. Compared with the incisor and canine, the force on the molar is relatively vertical, and many molar related studies use vertical force [18, 27]. If there is lateral force, it should also be in buccal-lingual section, and this study evaluated mesial-distal section. Even if we change the position and direction of the load, we can still get the result similar to Fig. 4, because the general direction of occlusal force was downward.

Despite great variations in crown material properties, there were only minor differences in the alveolar bone. For a given load configuration, it appears that overall stress distribution within the tooth-bone complex was more influenced by geometry design of restoration (endocrown vs. post-core crown) than by composition (e.g. crown, post and core restorative material type). The endocrown showed a relatively smooth stress distribution in the root and the periodontal support tissue. This is largely due to two reasons: firstly, post-core crown applied extra-coronal retainer, while endocrown used intra-coronal retainer. Endocrown with intra-coronal retainer was more conducive to transfer the force to the wall of the pulp chamber and the periodontal tissue, rather than to the root canal wall. Secondly, endocrown geometrically reduced the rotation center of the crown restoration in comparison with the full crown (Fig. 1c and d, ring arrow). This also contributed to transfer the occlusal force to periodontal tissue.

The root canal after instrumentation (root canal or post preparation) is thinner and weaker than the rest of the tooth. Stress concentration at the post tip must therefore be regarded as most harmful. It is precisely in the area of concentrated stresses where differences were found (Fig. 4, the left peaks of SS post and MC post). e.g. stresses in the post tip around SS posts achieved dozens of times higher values than under endocrowns and twice higher values than under GF posts. In this specific zone, low elastic modulus restorative materials showed reduced stresses, which can be explained by the stress redistribution into the more flexible GF post and composite core. Stress concentration of endocrown in the root canal was relatively small, it is good to avoid the weak tip of the post. The molar post-core crown increased the risk of accidental root fracture. The highest MPS in the distal root canal occurred in molar restored with SS and MC posts. These types of restorations seem to be the least beneficial in molar teeth. Biacchi and Basting [31] found that molars with endocrowns are more fracture resistant than teeth restored with GF posts and cores and ceramic crowns. Taking into consideration the suitable stress distribution of endocrowns, minimal invasive preparation of tooth structures and no roots damage, these restorations can be recommended to use in molars.

Although MPS levels in the root and the periodontal tissue of molars restored with post-core crowns were higher than stress levels in the tooth with the endocrown. The higher stress peaks in the crowns were observed in the materials with higher elastic modulus [18]. Zr endocrown represented the one condition with a slightly greater amount of stresses concentration in the cavity inner wall when compared to the post-core crowns. Thus, this is regarded as a potential threat, knowing that remaining coronal tooth structure fractured. Lithium disilicate ceramic materials presented acceptable survival and fracture load as long as the material’s minimum thickness and the enamel adhesion are respected [32]. In the 4 mm endocrown, the cement layer is far away from the load point, which is a protection. But increasing the thickness of the prosthesis means reducing the tooth resistance, which needs a balance. On the other hand, experimental strength study by Forberger and Göhring [33], have shown no significant differences between teeth restored with posts and endocrowns in terms of fracture resistance. Under the analytical conditions of this study, the results were largely dependent on the Young’s modulus and Poisson ratio of the materials. However in reality there will be other dominating factors such as the bond strength, potential for micro-crack at the interface, fatigue damage potentials both for the hard tissues and the restorative materials. Further experimental studies and clinical trials are needed to validate the results of this FEA study.

## Conclusions

From this study, the following could be concluded:

The endocrowns reduced stress concentration for the root canal inner wall in comparison with the conventional post-core crown. Endocrown exhibited higher stresses occurred at the root outer surface, while post-core crowns showed increased stresses at the root canal inner wall, especially stainless steel and metal cast post. Molars restored with endocrowns are less prone to root fracture than those with posts. The stress distributions in root were similar for different crown materials and different occlusal clearance.

## Data Availability

The complete data and materials described in the research article are freely available from the corresponding author on reasonable request.

## Abbreviations

2D: two dimensional
3D: three dimensional
Zr: zirconia
LDRGC: lithia-disilicate reinforced glass ceramic
GF: glass fiber
SS: stainless steel
MC: metal cast
ETM: endodontically treated molars
FEA: finite element analysis
CAD: computer aided design
N: Newton
MPS: maximum principal stress
